# InP/ZnS quantum dots doped blue phase liquid crystal with wide temperature range and low driving voltage

**DOI:** 10.1038/s41598-020-75046-0

**Published:** 2020-10-22

**Authors:** Jiayue Tang, Fashun Liu, Mengli Lu, Dongyu Zhao

**Affiliations:** Key Laboratory of Bio-Inspired Smart Interfacial Science and Technology, Ministry of Education, School of Chemistry, Beihang University, Beijing, 100191 P. R. China

**Keywords:** Materials science, Nanoscience and technology

## Abstract

Blue-phase liquid crystals (BPLCs) are regarded as potential materials for the exploitation of next-generation optical devices due to the rapid response, wide viewing angle, and simple industrial production procedures. However, practical application of traditional BPLCs is limited by their narrow temperature range and high driving voltage. Herein, we demonstrated that doping of chiral molecular isosorbide hexyloxybenzoate (R811) into BPLCs is able to increase the temperature range. More importantly, addition of InP/ZnS quantum dots (QDs) with oleylamine surface groups could also effectively broaden the temperature range of the BPLCs further while decreasing the driving voltage, which is attributed to the quantum dot trapped by BPLCs lattice defect that reduces its free energy. Since the trapped quantum dot subsequently forms a local electric field under electric field, the effective electric field of the surrounding liquid crystal molecules is enhanced and the rotation of the liquid crystal molecules is accelerated. Specially, the temperature range is widened by 1.4 °C, and the driving voltage is reduced by 57%, under the optimal concentration of R811 and lnP/ZnS QDs. The accomplishment we proposed in this work is a prospective optimization which makes the practical application of blue phase liquid crystals one step closer.

## Introduction

Blue phase liquid crystals (BPLCs) often appear in liquid crystal systems with strong optical rotation^1^. In recent years, BPLCs have become promising candidate as the next-generation optical displays due to macroscopical isotropy that can achieve display without the surface treatment of the substrate, which significantly reduces fabrication processes and costs^2–4^. At the same time, its advantages such as microsecond response that eliminates the need for filters and visual compensation films makes it possible to replace traditional displays^5–9^. However, disadvantages of the narrow temperature range and the high driving voltage hinder the practical application of BPLCs^10^.

In order to solve these problems, researchers began to use nanomaterial doping as a solution^1,11–14^. It is reported that the nanoparticles might fill the disclination region formed by the double twisted arrangement of the BPLCs molecules in space, which stabilizes the arrangement of the blue phase space and the blue phase liquid crystal system^15^. Its macroscopic representation is that the stable temperature range is widened. Recently, it has been reported that spherical gold nanoparticles with a radius of about 3.7 nm are introduced into the small molecule BPLCs system^14^. They found that gold nanoparticles can stabilize the BPLCs and broaden the blue phase temperature range. The temperature range of the BPLCs is broadened from 0.5 to 5.0 °C. In 2016, Arshdeep team found that silica nanoparticles can widen the blue phase of the EBBA-CN hybrid system from 2.3 to 6.0 °C^16^. Although there have been some research advances in the field of nanomaterials and BPLCs, the types of nanomaterials are relatively simple and the performance of BPLCs still cannot reach the requirements.

As a special nanomaterial, quantum dots (QDs) have the advantages of nanomaterials and the electro-optical properties. Some studies have reported the influence of the size of modified CdSe and CdTe QDs nanoparticles on the electro-optical properties of nematic liquid crystals. It is found that the doping of these two QDs reduces the distortion elastic constant of liquid crystal. The threshold voltage is lowered. Those studies concluded that QDs with a size of 2.5–5.2 nm and a concentration of less than 2.0 wt% can significantly improve the electro-optic properties of liquid crystals. Increasing alkyl chain can reduce the threshold voltage^17^. Cordoyiannis et al. reported the effects of CdSe (~ 4.5 nm in diameter) on the stability of the blue phase liquid crystal phase in CE6 using the characteristics of nanoparticles. The results show that the temperature range of CE6 blue phase III widens with the increase of CdSe content, which can increase to 5–15 times of the original temperature range, while the blue phase II gradually disappears and the blue phase I basically does not change. The surface of the CdSe nanomaterial is modified with a hydrophobic group (oleamide and trioctylphosphine) or a hydrophilic group, and it is found that the hydrophilic group-modified nanomaterial has no effect of stabilizing the blue phase liquid crystal^18,19^. In 2011, Taushanoff et al. used solvent evaporation to introduce hydrophobic water-modified silica nanoparticles (~ 7 nm in diameter) into curved liquid crystal molecules. The results show that the temperature range of blue phase III widens with the increasing content of nanomaterials^20^.

Based on the advantages of QDs doping, in this paper, we doped InP/ZnS QDs (Fig. S1) into BPLCs, and studied the effects of the QDs on BPLC temperature range and driving voltage. According to our findings, this is the first time to use lnP/ZnS QDs to optimize the temperature range and driving voltage of BPLC. With the doping of QDs, the temperature domain of BPLCs is expanded by 1.4 °C, while the driving voltage is reduced by 57%. Compared with previous studies, the driving voltage is greatly reduced and the temperature range is also widened, which is advanced. This makes the practical application of BPLC a step closer.

## Results and discussion

Due to its narrow temperature range, BPLC limits its wide application, and its temperature domain is affected by the proportion of chiral substances in the preparation of BPLC. Therefore, in this study, we adjusted the concentration of chiral substances in order to find out the best temperature domain of BPLC first. The temperature range was obtained by observing the change in BPLC texture in Fig. S2 with a polarizing microscope, showing a typical blue phase liquid crystal texture.

**Figure 1:**
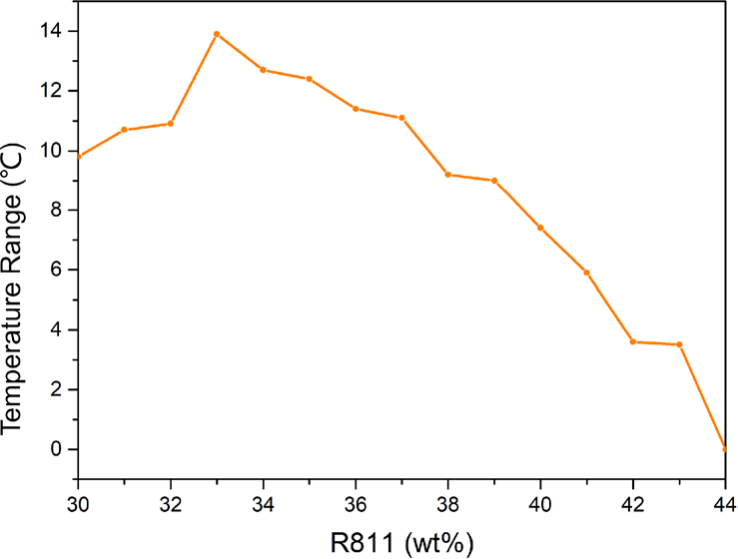
Variation of the temperature range of BPLCs with different concentrations R811.

**Scheme 1:**
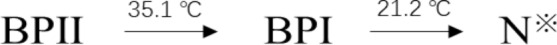
Phase transition temperatures of the LC material.

The effect of different concentrations of chiral material R811 on the temperature range of BPLC is shown in Fig. 1. When the concentration of R811 was increased from 30 to 33 wt%, the temperature range was increased from 9.8 to 13.8 °C. The phase transition temperature of BPLC is shown in Scheme 1. However, when the concentration of R811 continued from 33 wt%, the temperature range of BPLC began to decrease, after 44 wt%, blue phase liquid crystals no longer appeared, which may be caused by an excess of R811.

**Figure 2:**
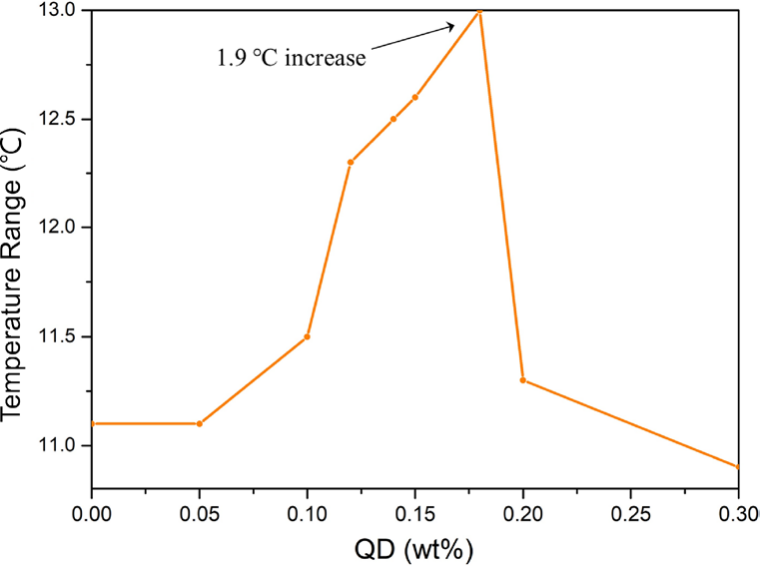
Variation of the temperature range of BPLCs with different doping concentrations of InP/ZnS QD.

In order to investigate the influence of InP/ZnS quantum dots with different concentrations on the temperature range and driving voltage of BPLC (RLC7011/R811 = 35/65), InP/ZnS QDs with different concentrations were dispersed into the matrix to prepare samples. The transmission electron microscope of the quantum dot is shown in Fig. S4–S6 in the Electronic Supplementary Material (ESM) We found that when the QDs concentration increases from 0 to 0.18 wt%, the temperature range gradually increases. As shown in Fig. 2, the maximum temperature is 13 °C at 0.18 wt%, which is 1.9 °C higher than that of pure BPLC. The typical BPLC texture is obtained as Fig. S3 with a polarizing microscope, showing a typical blue phase liquid crystal texture.

**Scheme 2:**
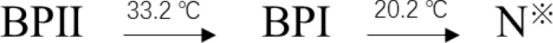
Phase transition temperatures of BPLC/QDs mixture.

The phase transition temperature of BPLC is shown in Scheme 2. When the content of QDs reaches 0.18 wt%, the concentration of QDs will continue to increase, and the temperature range will decrease rapidly. The mechanism of InP/ZnS QD broadening the temperature range of blue phase liquid crystal may be that the quantum dots in free motion can be trapped by defects in BPLC, and then the quantum dots self-assembly occupies the defects in the blue phase lattice, which makes the volume around the defects compressed and the free energy reduced. The macroscopic performance is that the temperature range of blue phase liquid crystal becomes wider^21^.

**Figure 3:**
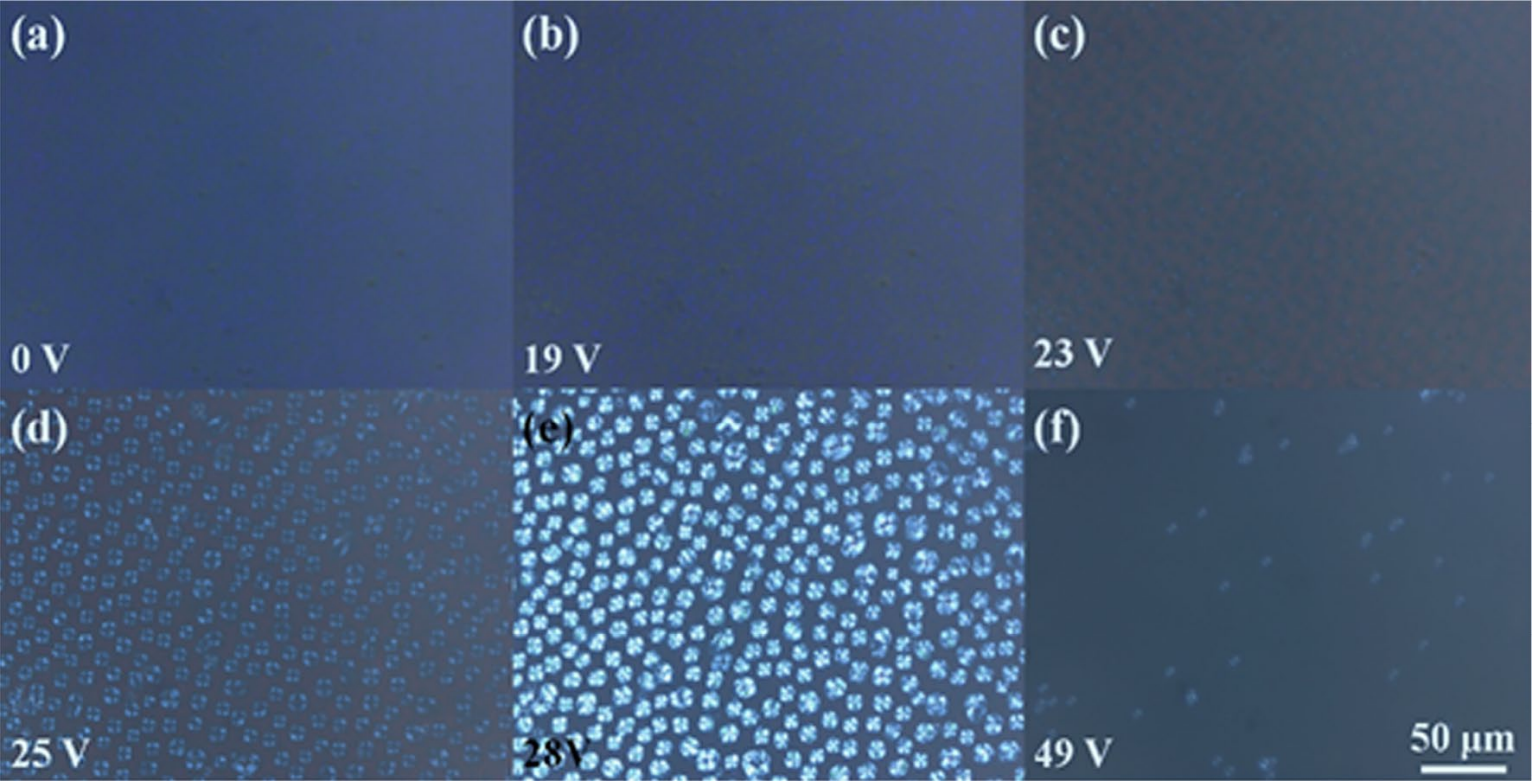
POM images of the pure BPLC under electric field. (**a**) 0 V. (**b**) 19 V. (**c**) 23 V. (**d**) 25 V. (**e**) 28 V. (**f**) 49 V.

The driving voltage is related to the energy consumption and cost of the liquid crystal product, as well as its own practicability and safety. It is an important indicator for selecting a liquid crystal device. Reducing the driving voltage can significantly improve the electro-optic performance of the liquid crystal device, reduce the power consumption of the display, and improve safety. Thereafter, in order to study the influence of the quantum dots on the BPLC driving voltage, a voltage was applied to the above BPLC, and the texture was observed by a polarizing microscope. Figure 3 shows the change in texture of a voltage applied to a BPLC without quantum dots.

**Figure 4:**
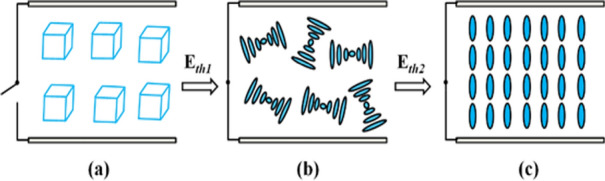
Schematic diagram of arrangement of blue phase liquid crystal molecules under the action of an electric field. (**a**) blue phase lattice, (**b**) focal cone arrangement, (**c**) vertical arrangement.

When the voltage reaches about 25 V, the transmittance of blue phase liquid crystal gradually decreases under the action of an electric field This may be caused by its conversion from the blue phase to the cholesteric phase (as shown in Figs. 3c–e and 4a,b). Continue to increase the electric field strength, the helical structure of the cone texture is gradually released. When reaching 49 V, the blue phase liquid crystal molecules gradually reach vertical alignment and exhibit a field-induced nematic phase. At this time, the liquid crystal molecules are arranged perpendicular to the glass substrate (as shown in Figs. 3f, 4c, and the transmittance increases. The voltage of the blue phase liquid crystal converted into the focal conic state is about 25 V, and the voltage of the field-induced nematic phase texture is 49 V. These two threshold voltages are the targets of the experiment. After that, we tested the driving voltage of the BPLC after the addition of the quantum dots.

**Figure 5:**
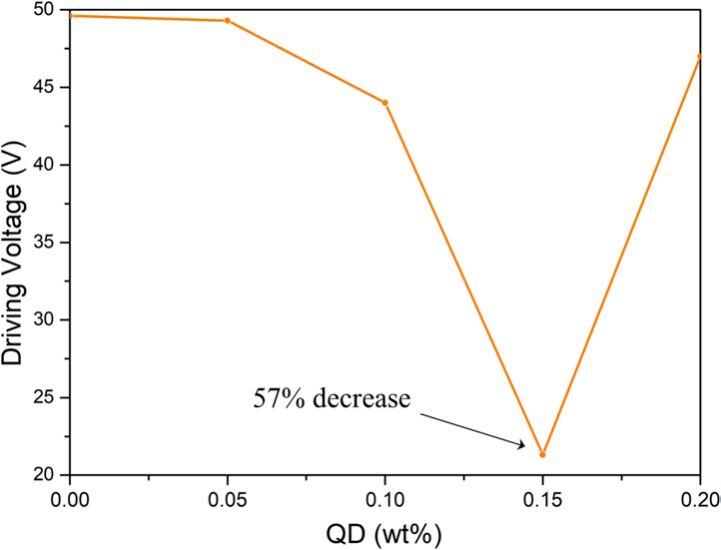
Variation of the driving voltage of BPLCs with different doping concentrations of InP/ZnS QD.

The driving voltage of BPLC with different quantum dots doping concentration was tested by the same method. As shown in Fig. 5, it can be seen that the addition of the quantum dots has a significant function of reducing the driving voltage. When the quantum dots concentration is 0.15 wt%, the transition is performed. The drive voltage for the nematic state is reduced by a maximum of 57%. At this concentration, the temperature range can also be widened by 1.4 °C.

**Figure 6:**
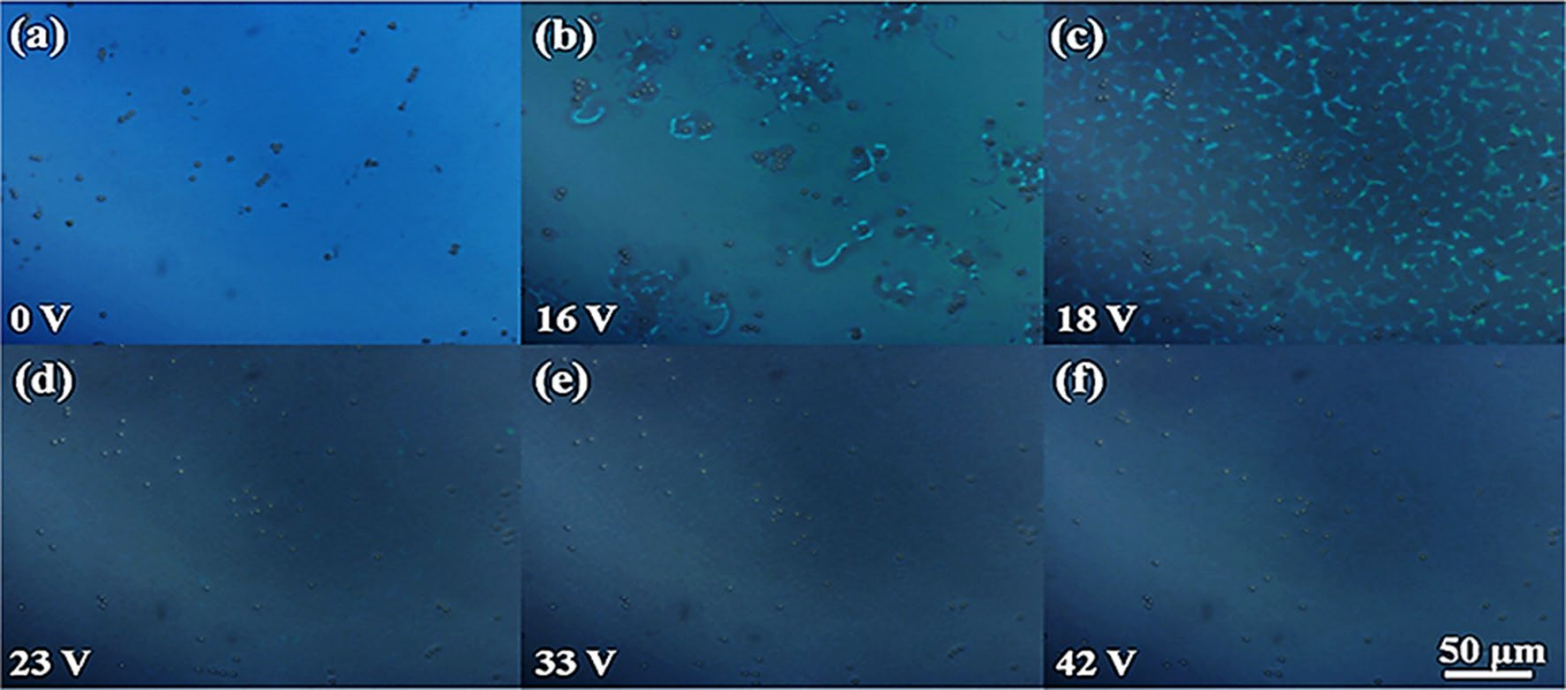
POM images of the BPLC/QDs under electric field. (**a**) 0 V, (**b**) 16 V, (**c**) 18 V, (**d**) 23 V, (**e**) 33 V, (**f**) 42 V.

In order to further understand the decrease in driving voltage, we observed the POM images of the sample under an electric field. Figure 6 shows the texture of liquid crystals at different electric field strengths at a quantum dot concentration of 0.15 wt%. It can be clearly observed that the focal conic state of BPLC does not appear, as in other samples with quantum dot concentrations shown in Fig. S4–S6 in the Electronic Supplementary Material (ESM).

**Figure 7:**
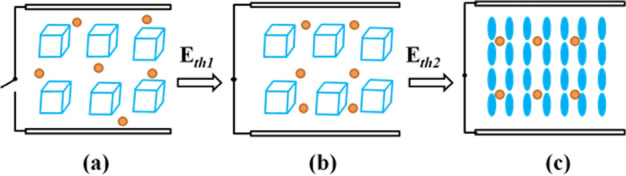
Schematic diagram of alignment of liquid crystal molecules of BPLC/QDs composite under the action of electric field: (**a**), (**b**) blue phase lattice. (**c**) vertical arrangement.

Based on the above POM images, we draw a schematic diagram of its phase transition. Figure 7 shows typical texture changes after the addition of quantum dots. Due to the protection of the oleylamine on the surface of the quantum dot, the focal phase of the blue phase liquid crystal converted into the field-induced nematic phase does not appear under the action of the electric field, as shown in Figs. 3 and 4b, which is beneficial to the application of blue phase liquid crystal in the display field. At the same time, InP/ZnS QD is a semiconductor material, therefore a polarized electric field can be formed under the action of an external electric field to enhance the effective electric field around it. Meanwhile, the quantum dots can adsorb the charged impurity ions to weaken the shielding effect, thereby greatly reducing the driving voltage of blue phase liquid crystal.

## Conclusion

In summary, we report a BPLC consisting of SLC7011 and R811, which is stabilized by InP/ZnS quantum dots. The POM study shows that when the concentration of R811 is 34 wt% and the doping concentration of quantum dots is 0.18 wt%, the temperature range of BPLC can be extended by about 1.9 °C; when the doping concentration of quantum dots is 0.15 wt%, the temperature range of BPLC can be extended by about 1.4 °C, while also reducing the voltage converted to a nematic state by 57%. At the same time, the above changes can be repeated after cooling and stopping the voltage and can last for months, which proves that the quantum dots to the BPLC is stable. Theoretically, this is usually thermodynamically stable, because quantum dots can fill in the line defects which free energy are high. Our research is expected to pave the way for the development of BPLC/QDs composites with a wide temperature range, low drive voltage and good stability, making the practical application of BPLC a step closer.

## Experimental

SLC7011 used in this experiment is from Hebei Shijiazhuang Chengzhi Yonghua liquid crystal material Co., Ltd., Δn = 0.15, Δε = 17.7 at 25 °C; chiral compound: R811 is from Tsinghua yawang liquid crystal Co., Ltd. InP/ZnS quantum dots (the surface group is oleamine) used in this experiment are from Suzhou xingshuo quantum dot company. All reagents used in this experiment were of analytical grade without further purification.

### Preparation of blue phase liquid crystal

The chiral compound R811 was separately added to the nematic liquid crystal SLC7011 in different proportions, and ultrasonically dissolved to obtain a mixture of SLC7011 and R811.

### Preparation of BPLC/QDs composite materials

A certain amount of BPLCs was weighed out, and a certain volume of QDs was taken out from a QDs/n-hexane solution having a concentration of 5 mg/mL and added to the BPLC system, and the ultrasonic dispersion was uniform. The above-mentioned QDs/BPLCs compound was ultrasonicated for 1 h, and then volatilized to a n-hexane solvent at room temperature to obtain quantum dot/blue phase liquid crystal composites in different ratios.

### Characterization

The Inp/ZnS was characterized by a jeol-jem-2100f. transmission electron microscope (TEM). The sample was subjected to TEM analysis at an acceleration voltage of 200 kV. The sample was obtained by evaporating a drop of diluted toluene solution from a nuclear power source to a carbon coated copper TEM grid. A typical TEM image of lnp/ZnS is shown in Fig. S1 in the Electronic Supplementary Material (ESM).

The BPLC/(InP/ZnS) composite is a capillary filled box consisting of two parallel indium tin oxide coated transparent glass plates. A film having a thickness of 10 μm was prepared on an OLYMPUS BX51 polarizing microscope, and the texture and temperature range of BP were measured by a polarizing microscope (POM). All microscope pictures are taken in reflection mode in the measurement, all samples are first heated to an isotropic phase of 35.0 °C, and cooled to room temperature at a rate of 0.5 °C per minute. When measuring the driving voltage, an alternating current with a frequency of 100 Hz was applied to the anti-parallel liquid crystal cell (with composite or pure blue phase liquid crystal), the waveform is a square wave, and the change of the texture under voltage was observed by a polarizing microscope to measure. Its drive voltage. In the process of testing the driving voltage, keep the temperature of the sample above 30° to ensure that all samples are blue phase.

## Supplementary information

Supplementary Information.
