# Supplementary material for: InP/ZnS quantum dots doped blue phase liquid crystal with wide temperature range and low driving voltage
Source: Sci Rep. 2020 Oct 22;10:18067. doi: 10.1038/s41598-020-75046-0 (PMC7582920; doi:10.1038/s41598-020-75046-0)
Supplement: Supplementary file 1 — Supplementary Information. [file 41598_2020_75046_MOESM1_ESM.doc]

Electronic Supplementary Material

| **InP/ZnS Quantum dot doped blue phase liquid crystal with wide temperature range and low driving voltage** |
| --- |
| Jiayue Tang, Fashun liu, Mengli Lu, Dongyu Zhao*  *Key Laboratory of Bio-Inspired Smart Interfacial Science and Technology, Ministry of Education, School of Chemistry, Beihang University, Beijing 100191, China*  E-mail of corresponding author：zhaodongyu@buaa.edu.cn  **Figure S1** TEM images of InP/ZnS quantum dots. |


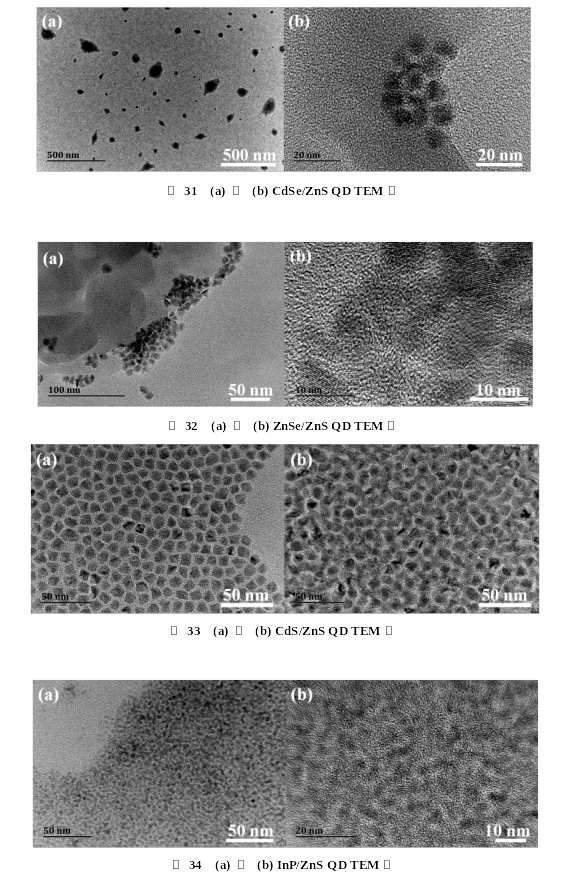


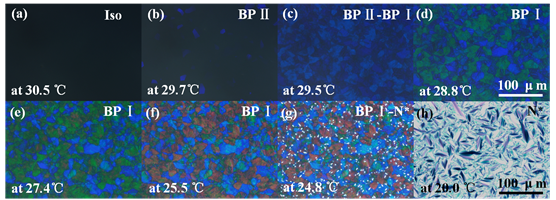


**Figure S2** POM images of BPLCs at different temperatures, with a cooling rate of 0.5℃/min.


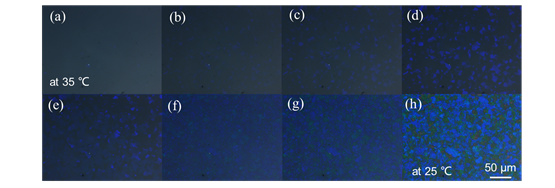


**Figure S3** POM images of BPLC with 0.05wt% InP/ZnS quantum dots at different temperatures, with a cooling rate of 0.5℃/min.


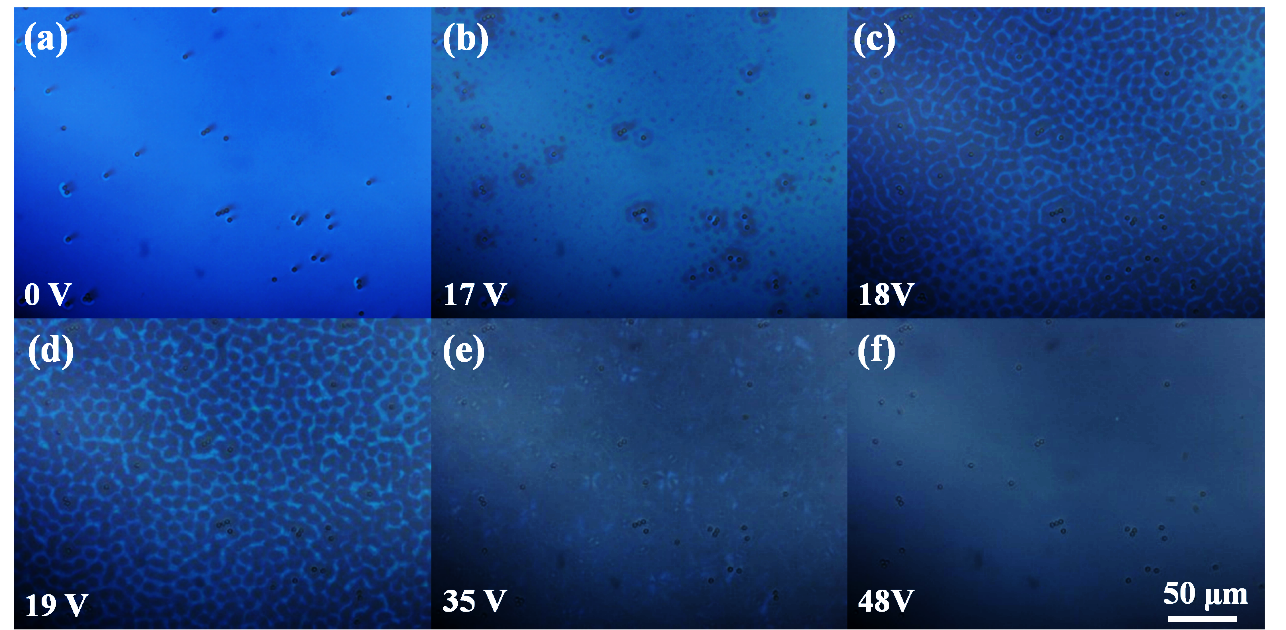


**Figure S4** POM images of BPLC with 0.05wt% InP/ZnS quantum dots under electric field. (a) 0 V, (b) 17 V, (c) 18 V, (d) 19 V, (e) 35 V, (f) 48 V.


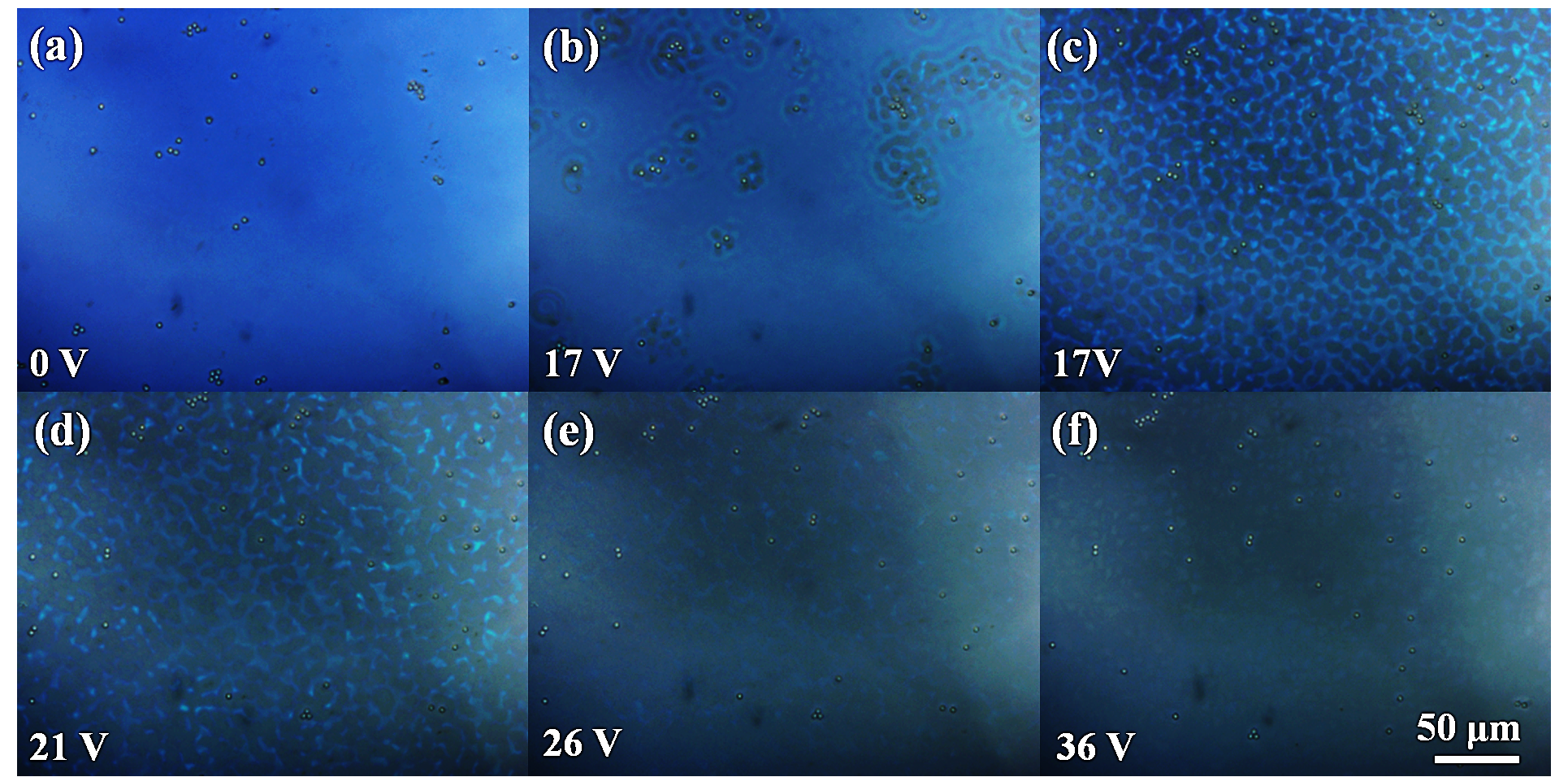


**Figure S5** POM images of the BPLC with 0.1 wt% InP/ZnS quantum dots under electric field. (a) 0 V, (b) 17 V, (c) 17 V, (d) 21 V, (e) 26 V, (f) 36 V.


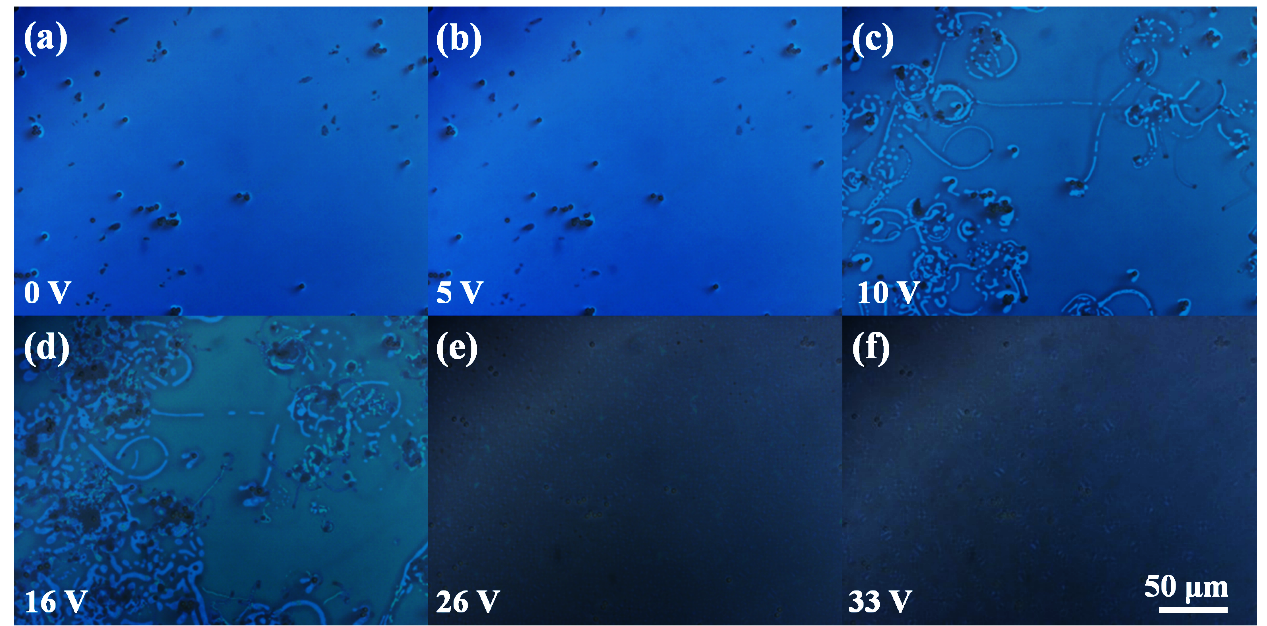


**Figure S6** POM images of the BPLC with 0.2 wt% InP/ZnS quantum dots under electric field. (a) 0 V, (b) 5 V, (c) 10 V, (d) 16 V, (e) 26 V, (f) 33 V.

|  |  |
| --- | --- |
| Corresponding author E-mail：zhaodongyu@buaa.edu.cn | |
